# Serro 2 Virus Highlights the Fundamental Genomic and Biological Features of a Natural Vaccinia Virus Infecting Humans

**DOI:** 10.3390/v8120328

**Published:** 2016-12-10

**Authors:** Giliane de Souza Trindade, Ginny L. Emerson, Scott Sammons, Michael Frace, Dhwani Govil, Bruno Eduardo Fernandes Mota, Jônatas Santos Abrahão, Felipe Lopes de Assis, Melissa Olsen-Rasmussen, Cynthia S. Goldsmith, Yu Li, Darin Carroll, Flavio Guimarães da Fonseca, Erna Kroon, Inger K. Damon

**Affiliations:** 1 Coordinating Center for Infectious Diseases, Centers for Disease Control and Prevention (CCID/CDC), Atlanta, 30329-4027 GA, USA; dtt4@cdc.gov (G.L.E.); ssammons@cdc.gov (S.S.); mfrace@cdc.gov (M.F.); dgovil@cdc.gov (D.G.); mOlsen-Rasmussen@cdc.gov (M.O.-R.); cgoldsmith@cdc.gov (C.S.G.); lay4@cdc.gov (Y.L.); dcarroll@cdc.gov (D.C.); 2 Department of Microbiology, Universidade Federal de Minas Gerais, Belo Horizonte, MG CEP 31270-901, Brazil; brunofmota@gmail.com (B.E.F.M.); jonatas.abrahao@gmail.com (J.S.A.); felipelopesassis@gmail.com (F.L.d.A.); fdafonseca@icb.ufmg.br (F.G.d.F.); kroone@icb.ufmg.br (E.K.)

**Keywords:** vaccinia virus, genome, virulence, outbreak, poxvirus, public health

## Abstract

Vaccinia virus (VACV) has been implicated in infections of dairy cattle and humans, and outbreaks have substantially impacted local economies and public health in Brazil. During a 2005 outbreak, a VACV strain designated Serro 2 virus (S2V) was collected from a 30-year old male milker. Our aim was to phenotypically and genetically characterize this VACV Brazilian isolate. S2V produced small round plaques without associated comets when grown in BSC40 cells. Furthermore, S2V was less virulent than the prototype strain VACV-Western Reserve (WR) in a murine model of intradermal infection, producing a tiny lesion with virtually no surrounding inflammation. The genome of S2V was sequenced by primer walking. The coding region spans 184,572 bp and contains 211 predicted genes. Mutations in envelope genes specifically associated with small plaque phenotypes were not found in S2V; however, other alterations in amino acid sequences within these genes were identified. In addition, some immunomodulatory genes were truncated in S2V. Phylogenetic analysis using immune regulatory-related genes, besides the *hemagglutinin* gene, segregated the Brazilian viruses into two clusters, grouping the S2V into Brazilian VACV group 1. S2V is the first naturally-circulating human-associated VACV, with a low passage history, to be extensively genetically and phenotypically characterized.

## 1. Introduction

*Vaccinia virus* (VACV) is a poxvirus that has played a remarkable role in the history of science as the agent used to eradicate smallpox, the most deadly disease since the beginning of civilization [1]. During the 1980s, VACV became the center of a new focus as possibilities emerged for its use in building expression vectors and recombinant vaccines [2]. Now, it is being used extensively for constructing recombinant vaccines for cancer and infectious diseases [2,3]. In the last decade, fears of smallpox reintroduction, emergence, and/or reemergence of zoonotic poxviruses have driven a renaissance in research addressing poxviruses zoonoses, vaccine design, and antiviral intervention [4]

VACV, as all members of the *Poxviridae* family and *Orthopoxvirus* (OPXV) genus, is characterized as a complex virus having a large brick-shaped or ovoid virion [4,5]. VACV is morphologically indistinguishable from other OPXV including *Variola virus* (VARV), *Cowpox virus* (CPXV), and *Monkeypox virus* (MPXV). VACV replicates in the cytoplasm and can grow in vitro in many cell lines [4,5]. During the replication cycle, VACV produces infectious viral particles with different stages of maturation; the intracellular mature virus (IMV), the extracellular enveloped virus (EEV), and the cell-associated enveloped virus (CEV) [6]. Like other OPXV, VACV has a linear double-stranded DNA genome that encodes approximately 200 genes, which are distributed into a central region containing conserved genes involved in virus replication and an inverted terminal region (ITR), located at both termini containing, usually, less conserved genes related to the mechanisms of virus-host interactions, thus reflecting the co-evolution process between poxviruses and their many hosts [7].

VACV is the most studied species among poxviruses and has been used as the prototype virus to elucidate many aspects related to the biology, immune response, pathogenesis, host evasion, and phylogenetics of the OPXV. In spite of so much knowledge regarding the biology of this virus, its origin and reservoir remain uncertain [1,2,3,4]. Recently, we and others have described this virus as naturally circulating in a zoonotic cycle involving bovine herds and dairy workers in Brazil [8,9,10,11,12,13,14,15]. Although some studies suggest that rodents could represent the natural reservoir of VACV in Brazilian forests [12,16,17], historically, the virus was thought to be restricted to the laboratory environment. As a result, vaccine escape has been hypothesized to account for the origin of the Brazilian VACV (BVV) [8,18,19,20]. A recent study presented a close phylogenetic relationship between S2V, VACV-IOC (a smallpox vaccine strain used in Brazil) and the presumed ancestor-like horsepox virus (HSPV) [21].

However, genetic comparison of the limited DNA sequence data collected thus far has been unable to sustain this hypothesis with regard to members of the more virulent group of BVV (group 2), suggesting that multifactorial events may best explain the genetic diversity present among the strains now circulating [19,20,21]. In vivo studies have shown that different BVV strains are quite different regarding virulence on intranasal infection in BALB/c mice [22]. Another approach to test the virulence of OPXV based on inoculation of the ear pinnae of mice has been described previously and yielded similar results [23].

Outbreaks of an exanthematous disease affecting dairy cattle and dairy workers were sporadically reported in Brazil during smallpox eradication [24,25]. Similar outbreak incidents were reported in many countries across India and Latin America [26,27]. Since 1999, VACV has been frequently and consistently isolated in association with outbreaks having substantial impact on local economies [8,9,10,11,12,13,14,15]. Retrospective epidemiological data have revealed that 100% of symptomatic patients develop several classical flu-like symptoms and cutaneous lesions after direct contact with infected cows. Clinicians have had difficulty diagnosing and managing these infections and have raised their concerns to the board of public health [28,29].

During an outbreak in 2005, a clinical sample was collected from a 30-year old male milker presenting with lesions on his hands and associated lymphangitis, prominent axillary lymphadenopathy and several flu-like symptoms [28]. The patient had not been previously vaccinated against smallpox and peripheral blood mononuclear cell (PBMCs) extracted from him approximately 10 days after the onset of infection showed a remarkable down-modulation of T and B cells, as well as production of interferon-γ (IFN-γ), after restimulation with VACV antigens [28]. A virus was isolated from vesicular material by standard methods [9,10,11,12] and initial molecular diagnostics were performed by the polymerase chain reaction (PCR) amplification of OXPV single genes (*TK*, *VGF*, *HA*, *E3L*, *K3L*, *B18R*, and *B8R*) following protocols described elsewhere [9,10,11,12]. The virus was named Serro 2 virus (S2V) based on the geographic site of discovery and because it was isolated from a human presenting an infection after contact with an infected cow (the source of Serro 1 virus). Subsequently, analysis of a large portion of the genome grouped this virus with other natural circulating virus and with a vaccine strain used in Brazil (VACV-IOC). In this study, we present the first extensive phenotypic and molecular characterization of a BVV isolated from a human. In addition to biological and molecular comparisons we explore issues regarding virulence, host interactions, and drug susceptibility.

## 2. Materials and Methods

### 2.1. Laboratory Assays

#### 2.1.1 Virus Isolation and Clonal Expansion

S2V was isolated from vesicle liquid collected from a 30-year old milker. The liquid was collected in 1 mL insulin syringes using 0.45 mm × 13 mm needles and kept on ice until arrival at the laboratory. The vesicular content was mixed with saline containing a two-fold antibiotic concentration (25 g/mL fungizone, 500 U/mL penicillin, and 50 g/mL gentamicin) to avoid contamination. The material was used to inoculate chorioallantoic membranes (CAMs) of nine-day old embryonated chicken eggs as described elsewhere [12]. After isolation from CAMs the virus was cloned by plaque purification under a 1% agarose overlay in Vero cells. One isolated plaque was collected, re-cloned on CAM and after clonal expansion the virus was designated S2V. S2V stocks were propagated on BSC40 cells following standard procedures. Virus was purified by Joklik’s protocol [30].

#### 2.1.2. Plaque Phenotype Assay

Plaque assay was carried out in BSC40 cells by using standard procedures described elsewhere [9]. Each virus was diluted in Roswell Park Memorial Institute (RPMI) medium supplemented with 2% fetal bovine serum to achieve ~20–60 plaque forming units (PFU) per well [12]. The following viruses were used for comparison with S2V: VACV-WR (VACV-Western Reserve), VACV-LIS (VACV-Lister), VACV-NYCBH (VACV- New York City Board of Health) Acambis 2000, VACV-NYBH Dryvax, VACV-IHDJ, and HSPV (*Horsepox virus* strain MNR-76). Plaques and comets were visualized by staining the cell monolayer following an immunohistochemical protocol [31]. Cells were fixed and exposed to polyclonal rabbit anti-VACV antibody (Virostat, Portlamd, ME, USA). After incubation with goat anti-rabbit horseradish peroxidase (HRP)-conjugated antibodies (Kirkegaard & Perry Laboratories, Gaithersburg, MD, USA) plaques and comets were visualized by addition of TrueBlue peroxidase substrate (Kirkegaard & Perry Laboratories). Other poxviruses used during this study are from the Centers for Disease Control and Prevention (CDC) collection and were propagated, purified, and titrated following standard procedures [9,10,11,12]. VACV-NYCBH Dryvax used here is a commercial vaccine reconstituted in saline solution as indicated by the (Wyeth Laboratories, Marietta, PA, USA) and was used directly from the original container without undergoing propagation or purification.

#### 2.1.3. One-Step Growth Curve

A one-step growth curve was carried out using confluent BSC40 monolayers in six-well plates. Cells were infected with 5 PFU/cell of S2V and VACV-NYCBH Acambis-2000 (either purified virus or crude preparation). At various times after the infection (3, 6, 12, 24, 36, and 48 h) the infected cell medium was removed and stored at 4 °C. In order to neutralize mature virus (MV) particles that could be present in the medium we used anti-MV J2D5 antibody [31]. Cells were washed with 1 mL of 1 × phosphate-buffered saline (PBS), lysed by freezing and thawing, harvested, sonicated, and stored at −80 °C. Virus yields were then determined by standard titration protocols on BSC40 cell monolayers. The IMV and EEV titrations were incubated for 72 h (dilutions from 10^−3^ to 10^−8^).

#### 2.1.4. Electron Microscopy

Monolayers of BSC40 cells were infected with S2V at 1 PFU/cell. After 16 h, cells were scraped from the flask, centrifuged, and fixed with PBS containing 2.5% glutaraldehyde. Cells were post-fixed in 1% osmium tetroxide, dehydrated, and embedded in a mixture of Epon-substitute and Araldite epoxy resins (Sigma-Aldrich, Saint Louis, MO, USA).

#### 2.1.5. In Vivo Analysis

Female specific-pathogen-free BALB/c mice were obtained from the Jackson Laboratory (Bar Harbor, MN, USA) and were used between six and eight weeks of age. Animals were anesthetized with an intraperitoneal injection of ketamine (100 mg/kg of body weight) and xylazine (10 mg/kg) diluted in sterile PBS. Following, 10 microliters of PBS containing 10^5^ PFU or 10^6^ PFU of VACV-S2V, or 10^6^ PFU of VACV-NYCBH Acambis-2000, or 10^5^ PFU of VACV-WR were inoculated intradermally in both ears as described previously [23]. For in vivo studies, VACV-WR and VACV-NYCBH Acambis-2000 were chosen for biological comparison. VACV-WR was chosen because this strain is the laboratory prototype widely used in animal studies, and VACV-NYCBH Acambis-2000 because it is a licensed vaccine used currently in USA specific populations such as military and health professionals. Control animals received PBS only. A lower dose was used for VACV-WR to avoid distress in animals, since this strain is more virulent in mice [22]. These groups of animals (*n* = 5) were inspected daily for any clinical signs of disease (decrease feeding and activity and weight loss) and lesion diameter were measured with a micrometer until day 28 post-infection (p.i.) and then euthanized. In a parallel experiment, groups of animals (*n* = 5) were infected in the same way as above, and then euthanized at day 5 p.i. and the ears removed for virus quantification. All animal experiments were approved by the CDC Institutional Animal Care and Use Committee (IACUC), USA (Protocol number: 1493-A3). Only the lesion border was measured and not the induration area.

#### 2.1.6. Viral Titration

At the time points indicated, animals were humanely euthanized by cervical dislocation under deep anesthesia and the ears pinnae were removed with a surgery scissor. The samples were homogenized in 1 mL of PBS using a 2000Geno/Grinder (SPEX CertiPrep, Metuchen, NJ, USA). Homogenates were sonicated for three minutes at 40% amplitude, frozen and thawed twice (−80/37 °C), sonicated again with the same conditions, and then serially diluted in RPMI 2% fetal calf serum (FCS). Dilutions were added to monolayers of BSC40 cells seeded in six-well plates, incubated for one hour at 37 °C and 5% CO_2_ atmosphere, then 2 mL of the medium were added to each well and further incubated at the same conditions for 48 h. After that time, cells were stained with a crystal violet solution (0.5% crystal violet, 10% ethanol, and 1% paraformaldehyde) for 20 min, washed again and the viral plaques were counted. The number of plaques was multiplied by the reciprocal of the sample dilution and converted to PFU/ear.

### 2.2. Molecular Characterization

#### 2.2.1. DNA Extraction and Sequencing Protocols

Stocks of purified S2V were used for DNA extraction using a BioRobot EZ1 workstation (Qiagen, Hilden, Germany), and the protocol to purify genomic DNA from tissue (Qiagen) then stored at 4 °C. Genomic DNA was then used as template for producing 20 overlapping PCR amplicons that span the viral genome. Templates were sequenced by primer walking both strands using Applied Biosystems (PE Biosystems, Foster City, CA, USA) Big-Dye 3.1 dye chemistry and ABI 3730XL automated DNA sequencers. Sequencing primers were synthesized by Integrated DNA Technologies (Coralville, IA, USA). Approximately 2580 reads were acquired, resulting in a nine-fold average redundancy at each base position. Chromatogram data were assembled using Seqmerge (Accelrys Inc., Madison, WI, USA), Phred/Phrap (Department of Genome Sciences of University of Washinton, Seattle, WA, USA) for base-calling and assembly, and Consed (Department of Genome Sciences of University of Washinton, Seattle, WA, USA) for sequence editing.

#### 2.2.2. Sequence Analysis and Alignment

Sequence annotation employed a locally-modified version of Poxvirus Orthologous Clusters software [32]. Genes were predicted using GeneMarkS [33] and Glimmer 2.02 [34]. They were tested for the presence of regulatory elements and assigned an initial annotation by comparison to other poxvirus gene databases, using BLASTP [35]. The remaining open reading frames (ORFs) were then verified by manual inspection. Genome alignments were generated with Mauve 2.4.0 software (University of Wisconsin–Madison, Madison, WI, USA) using default parameters and then hand-curated. The S2V genome sequence has been deposited in GenBank under accession number: KF179385. An ORF map is presented in the Figure S1.

S2V and several VACV previously isolated in Brazil were compared using a dataset of nine genes (*B5R*, *B8R*, *B19R*, *C6L*, *C7L*, *E3L*, *K1L*, *K2L*, and *K3L*) (see Table S1 and Table S2 for accession numbers) related to immune modulation strategies of the virus plus the *A56R* gene that encodes the viral hemagglutinin. VACV strain IOC, Lister Butantã, and both vaccine strains used in Brazil were also included in this analysis [20]. Genes were aligned in Geneious 8.1.4 [36] using ClustalW [37] and the Translation Align option with the BLOSUM cost matrix. A phylogenetic tree was estimated using the MrBayes plugin in Geneious with a Markov chain Monte Carlo (MCMC) length of five million, general time reversible (GTR) + I + G nucleotide substitution rate model, sampling every 1000 iterations and a burn-in length of one million [38]. Genomes and single gene sequences analyzed in this study are listed in Table 1.

## 3. Results

### 3.1. Virus Isolation and Phenotypic Characterization

**Figure 1:**
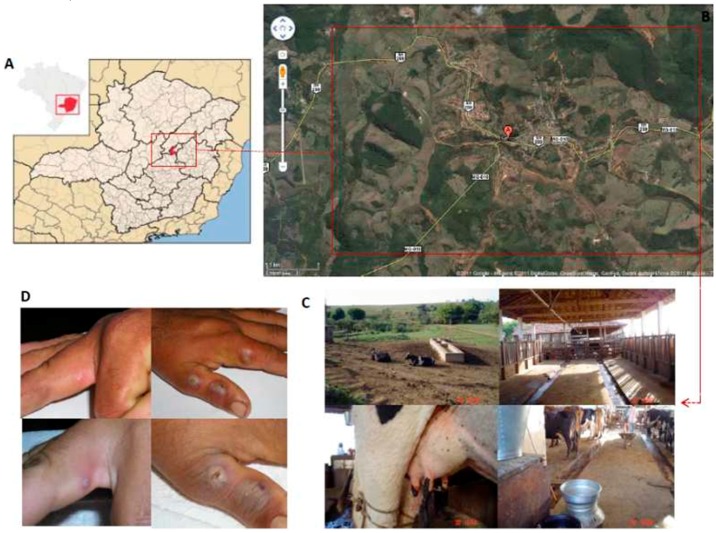
Location of Serro county Bovine Vaccinia (BV) outbreak area. (**A**) Brazil and Minas Gerais State maps showing Serro County (18°36’ S 43°22’ W) in red; (**B**) Panoramic view of the outbreak area showing relief topography and vegetation; (**C**) Typical milking farms landscape and infrastructure. *Vaccinia virus* (VACV) lesions on a cow udder and teats; (**D**) Lesion patterns and evolution in a primary VACV zoonosis infection. Vesicle lesion containing liquid from which S2V was isolated; vesicles evolved to pustules and ulcers with focal necrotic tissue. Areas of inflammation can be seen surrounding lesions.

S2V was isolated during a bovine vaccinia outbreak investigated in 2005 in the county of Serro, Minas Gerais State, Brazil (18°36 S 43°23′ W) (Figure 1A,B). The virus was isolated from a 30-year old male patient without previous anti-smallpox vaccination history (based on his age and lack of vaccination scar) that reported repeated direct contact with cows presenting lesions on teats and udders (Figure 1C). No VACV outbreaks were recorded in that region prior to 2005. The patient noticed skin lesions on his hands and fever. The presence of the exanthema (Figure 1D) was coincident with the development of peripheral lymphangitis and axillary lymphadenopathy. Rash development was concomitant with the development of flu-like symptoms including headache, myalgia and fever. CAMs infected with patient vesicular material showed typical OPXV cytopathic effect characterized mainly by the presence of white, non-hemorrhagic pocks suggestive of VACV (data not shown).

**Figure 2:**
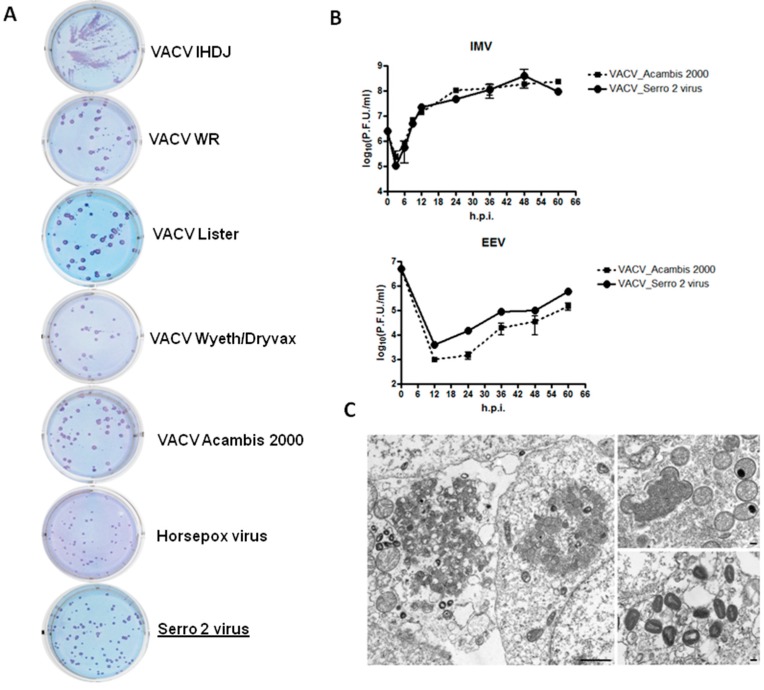
VACV strain Serro 2 virus (S2V) in vitro analysis. (**A**) Plaque morphology assay in BSC40 cells. For comparison, the assays were performed in parallel with a panel of vaccinia viruses from the Centers for Disease Control and Prevention (CDC) collection. Representative images of two independent experiments are shown; (**B**) One-step growth curves for S2V and VACV Acambis-2000. Monolayers of BSC40 cells were infected with 5 plaque forming units (PFU)/cell. At the indicated time points, cells were scraped and both cell associated viruses (CAV) and extracellular enveloped viruses (EEV) were collected and subjected to titration on the same cells. The results represent the average of two infected wells; (**C**) Electron microscopy of S2V-infected BSC40 cells. Typical poxvirus factories (left) contain nascent and intermediate particles, with mature viruses (MVs) within the cytoplasm. Higher magnifications of the particles show nascent (crescent-shaped) and intermediate (round-to-oval) forms within a virus factory (upper right) and MVs are found within the cytoplasm (lower right). Bars, 1 µm (left); 100 nm (upper and lower right).

To further characterize S2V phenotypically, plaque assays were conducted to determine plaque size, shape, and comet production. S2V exhibited a plaque phenotype in BSC40 cells (Figure 2A) that was visibly smaller than plaques produced by other selected VACV, including the vaccine viruses VACV-NYCBH Dryvax and Lister. Horsepoxvirus (HSPV strain MNR-76) produced slightly smaller plaques than S2V. In addition, S2V does not produce comet-shaped plaques as other VACV (Figure 2A).

The replication kinetic and growth properties of S2V under one-step growth conditions were characterized (Figure 2B). VACV-NYCBH Acambis-2000 was used for comparison. VACV-NYCBH Acambis-2000 is a Food and Drug Administration (FDA)-licensed clonal smallpox vaccine manufactured in cell culture and derived from VACV-NYCBH Dryvax vaccine, the smallpox vaccine previously used in the USA. VACV-NYCBH Acambis-2000 is known to be equivalent to VACV-NYCBH Dryvax in terms of cutaneous response rate, antibody response and safety [1,4]. Due to its clonal derivation from VACV-NYCBH Dryvax and similar response characteristics, VACV-NYCBH Acambis-2000 was used here as a surrogate for VACV-NYCBH Dryvax itself, which is known to contain a population of variable VACV clones [39]. Figure 2B shows the time course experiment with S2V and VACV-NYCBH Acambis 2000 viruses. The results revealed that in BSC40 cells the two viruses replicated with very similar kinetics producing relatively comparable amounts of IMV.

The next step was to quantify of the amount of virus released into the media, which is comprised mostly of EEV. Both S2V and VACV-NYCBH Acambis-2000 produced similar amounts of EV that increased after 12 h post-infection (h.p.i). The quantity of EEV produced by S2V and VACV-NYCBH Acambis-2000 was lower than the quantity of IMV produced under the same conditions indicating that S2V one step growth curve follows the classical poxvirus replication kinetics (Figure 2B).

Electron microscope images of the viral isolate (Figure 2C) show details of viral morphogenesis. Typical poxviruses nascent and intermediate forms are found within the virus factories, and IMVs are found at the edges of the virus factories and within the cytoplasm of the infected cells.

### 3.2. In Vivo Analysis

**Figure 3:**
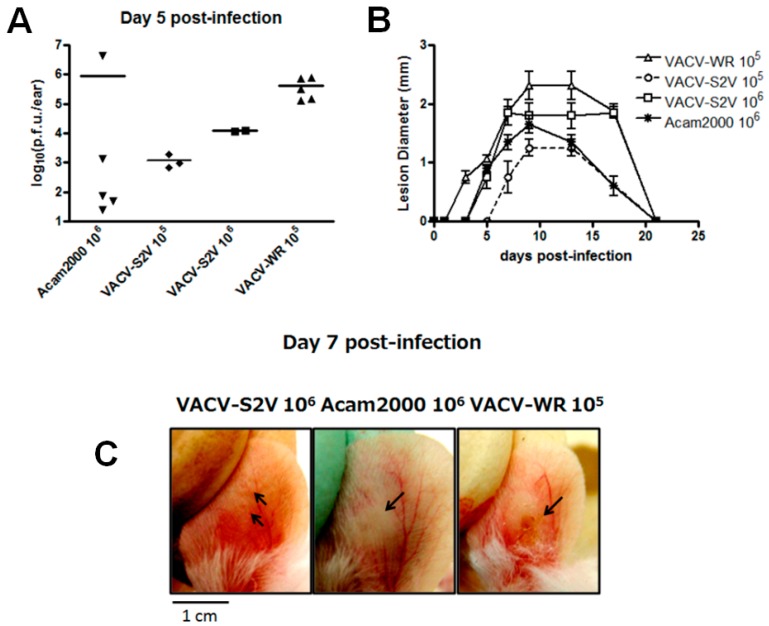
S2V in vivo analysis. In vivo intradermal infection in female BALB/c mice. Groups of five animals were infected with 10^6^ PFU/animal of Acambis-2000 (Acam2000), 10^5^ PFU/animal of Western Reserve (WR), and both 10^5^ and 10^6^ PFU/animal in the case of VACV-S2V. (**A**) At day 5 post-infection (p.i.), animals were humanely euthanized and ears were removed and processed for viral tritation in BSC40 cells; (**B**) Lesions diameter (in millimeters) in animals (*n* = 5) infected with the three VACV strains during 28 days post-infection; (**C**) Macroscopic examination of lesions in the ears of animals infected with the three VACV strains seven days post-infection. Only the higher dose is shown for VACV-S2V (10^6^ PFU). Note that the lesion caused by VACV-S2V has virtually no surrounding inflammation, in striking contrast with lesions caused by VACV-NYCBH Acambis-2000 and VACV-WR. The photos are representative of the group indicated (*n* = 5).

BALB/c mice were infected intradermally in the ear pinnae with S2V (10^5^ and 10^6^ PFU/ear). The peak mean titer was detected at day 5 p.i., regardless of the dose used. At this time point, the replication of S2V in ears of mice was about three logs lower than in the prototype strain VACV-WR using the same dose (10^5^ PFU) but was somewhat higher than in the vaccine strain Acambis-2000 (Figure 3A). Lesion diameter correlates well with the viral titration data (Figure 3B), with WR causing the largest lesion, followed by S2V and Acambis-2000 (all 10^6^ PFU). Lesions caused by S2V had a different appearance, with a reduced inflammation when compared to WR and Acambis-2000; WR produced lesions with a large area of surrounding induration (Figure 3C). None of the animals presented signs of generalized disease, consistent with previous data [22].

### 3.3. The S2V Genome

In this study the genome of S2V was sequenced (accession number: KF179385) and used for both whole genome and single gene comparative analyses as described above. We compared the S2V genome with VACV-NYCBH Acambis-2000, COP, and VACV-WR. The major differences between the S2V genome and other OPVX are shown graphically in Figure S1, which presents an ORF map of S2V. Comparison of the S2V (M35027) and VACV-COP (M35027), and VACV-NYCBH Acambis-2000 (AY313847) and VACV-WR (AY243312) genomes indicated that S2V gene structures and genome topography were very similar to other VACV. The coding region of the S2V genome is 184,572 bp long and contains 211 predicted genes with a CG content of 33.24%. The central region is typically conserved and five major deletions were found in the ITRs (for more information see Figure S1).

### 3.4. Comparison with Other VACV Strains

Driven by the possibility of deliberate VARV reintroduction and emergence of zoonotic poxviruses [40,41], recent research has centered attention on the OPXV host range, virulence, and immune protection. Since interest in these areas are mainly focused on the development of new diagnostic targets, safe VACV vaccines, and OPXV residual immunity, we further compared host immune response genes of S2V with those of other vaccinia viruses.

### 3.5. Genes Associated with Humoral Immune Response

**Figure 4:**
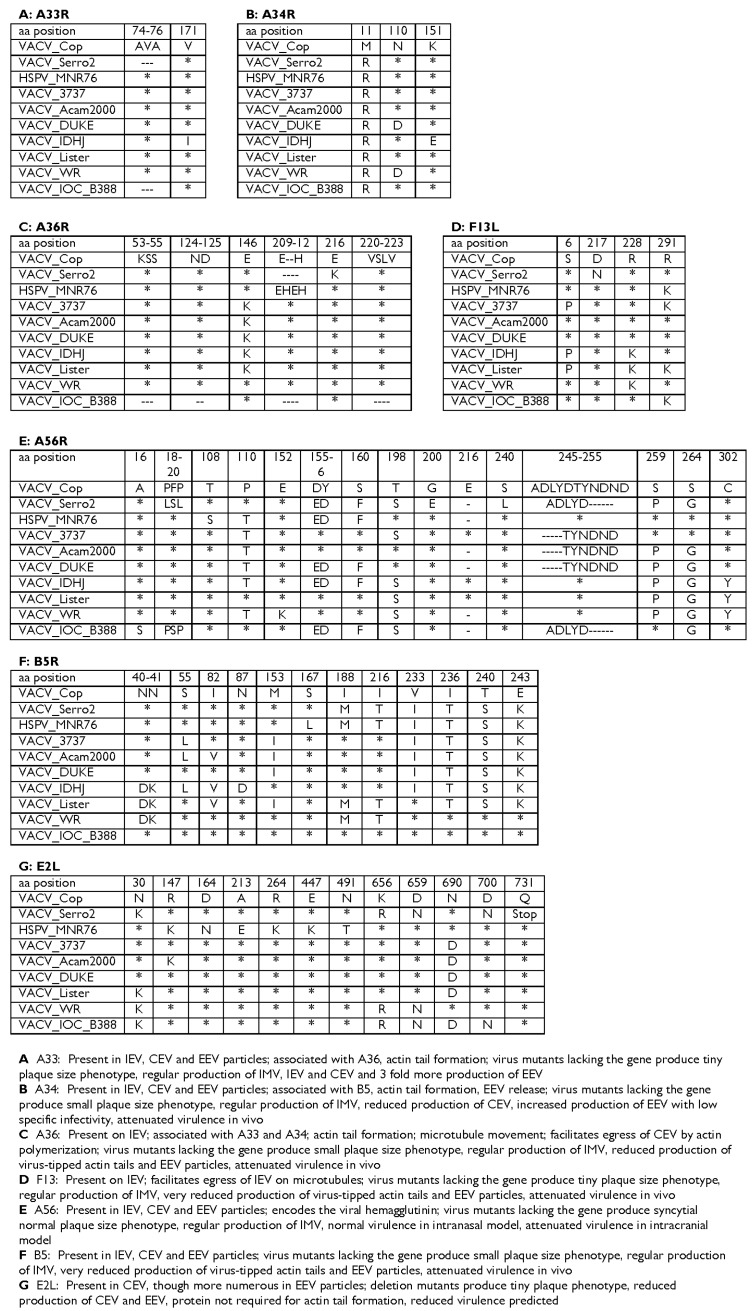
Comparison of amino acid alignments of the EEV genes associated with immune response in VACV strains, horsepox virus (HSPV), and S2V. Vaccinia Copenhagen (VACV-COP) was used as the reference sequence. Numbers at the top of each panel indicate the amino acid position in the VACV-COP protein sequence. The asterisks show conserved amino acids. The dashes show deleted amino acids.

A27L and H3L proteins, present in the MV, and A33R and B5R proteins, present in enveloped virus (EV) membranes, are examples of humoral immune response targets in that they are able to induce neutralizing antibodies in humans [42,43,44,45,46,47]. Genomic analysis revealed that all four immunodominant proteins are present in the S2V genome and are highly conserved in comparison with VACV-COP and other VACV strains. S2V presented some unique single nucleotide polymorphisms (SNPs) and also shared particular SNPs with VACV-WR, Acambis-2000, VACV-LIS, VACV-IOC, and HSPV (data not shown for A27L and H3L). Data from A33R and B5R are depicted in Figure 4. The S2V *A27L* gene is highly conserved and shares 100% identity with VACV-WR and HSPV (data not shown). Comparative analysis of *B5R* gene of S2V with VACV-LC16mO revealed that S2V does not share the premature stop codon seen in this Lister-derived Japanese strain. Recent literature demonstrates that the B5R product is a primary target for EV-neutralizing antibodies in vaccinated individuals [47,48,49,50]. Major neutralization sites have been identified within the B5R protein [47,48,49,50]. We compared these target regions among VACV and vaccine strains and found the S2V amino acid sequence to be identical to VACV-WR and VACV-COP. The *H3L* gene of S2V codes for an amino acid sequence identical to that of VACV-WR. Another protein found to be highly immunogenic among OPXV is the ATI protein which is the major protein present in A-type inclusion bodies [51]. The S2V genome retains the “*ati*” gene and the coding region showed unique substitutions and insertions and deletions (INDELs) that are typically found in only one group of BVV (i.e., Passatempo, GP2, and Araçatuba viruses) as described by Leite et al. [52]. The VAVC-IOC clone B388 was used here for comparisons genes with known relevance to immune response. However, it should be noted that VACV-IOC clone B141 is not identical to clone B388. B141 has the following differences: the gap in A36R at position 53–55 is not present, positions 155–156 and 160 in A56R are DY and S, respectively, and position 243 in B5R is K.

The more dramatic changes in the S2V genome in comparison with other VACV are five deletions that, together, comprise a region of approximately 10 kbp. These deletions occur in a region that, in VACV-COP, codes for several genes related to immune evasion, especially ORFs *C21L* to *C17L* that are the same as *B23R* to *B27R* and *B16R*. The proteins produced from ORFs *C21L* to *C17L* (in the left ITR) and *B23R* to *B27R* (in the right ITR) belong to the family of ankyrin-containing motif proteins, which includes eukaryotic proteins that regulate the complement system [53]. These proteins in OPXV also inhibit the alternative and classical pathways of complement activation, and contribute to virulence in the rabbit model of infection [2]. The ORF *B16R*, absent in the S2V genome, encodes a soluble form of the interleukin-1 receptor (IL-1R) that binds to host IL-1 and dampens the immune response normally elicited by this cytokine. This mechanism is believed to counteract the inflammatory response and is shown to influence virulence in the mouse model [54].

### 3.6. Immunomodulatory Genes and Virulence Factors

In order to develop hypotheses regarding virus–host interactions, we analyzed 17 genes involved in immune evasion or host range (Table 2) [55,56,57,58,59]. Several anti-apoptotic proteins encoded by S2V were also analyzed by amino acid alignment (Table 2). All genes were properly encoded by S2V and did not show premature stop codons or other dramatic differences (data not shown) when compared with other VACV strains. In addition, we have looked at four genes more closely, *E3L*, *K3L*, *B8R*, and *B19R*, which are related to the IFN response. These genes are present in the S2V genome and their structure appears to be intact and similar to that of VACV-COP and WR (Figure 4 and Table 2).

An interesting truncation was found in the *B13R* gene of S2V due to an early stop codon at amino acid position 117. B13R is a potent caspase-1 and caspase-8 inhibitor, which is not expressed by VACV strains Copenhagen, Tashkent, Lister, and VACV-NYCB Acambis-2000, due to mutations that lead to an N-terminal truncation of the protein in those genomes. The B13R product of S2V, like VACV-COP, is truncated by 219 amino acids compared to VACV-WR and HSPV. S2V, VACV-COP, and VACV-NYCB Acambis-2000 are also alike in that they are missing the first 10 codons of the *B13R* gene as compared to WR and HSPV. Only 116 amino acid in length, the protein is likely not expressed by S2V either. Another striking difference is present in the gene encoding the interleukin-1β (IL-1β) receptor homolog of S2V (*vIL-βR*); *B16R* in VACV-COP. The B16R homolog in S2V is similar to that of VACV-WR in its initial coding region; however, a large deletion of approximately 1440 bp and unique to S2V interrupts the coding region and creates an early stop codon, presumably resulting in premature truncation of the protein after 117 amino acids.

### 3.7. Targets of Antiviral Drug Therapies

**Figure 5:**
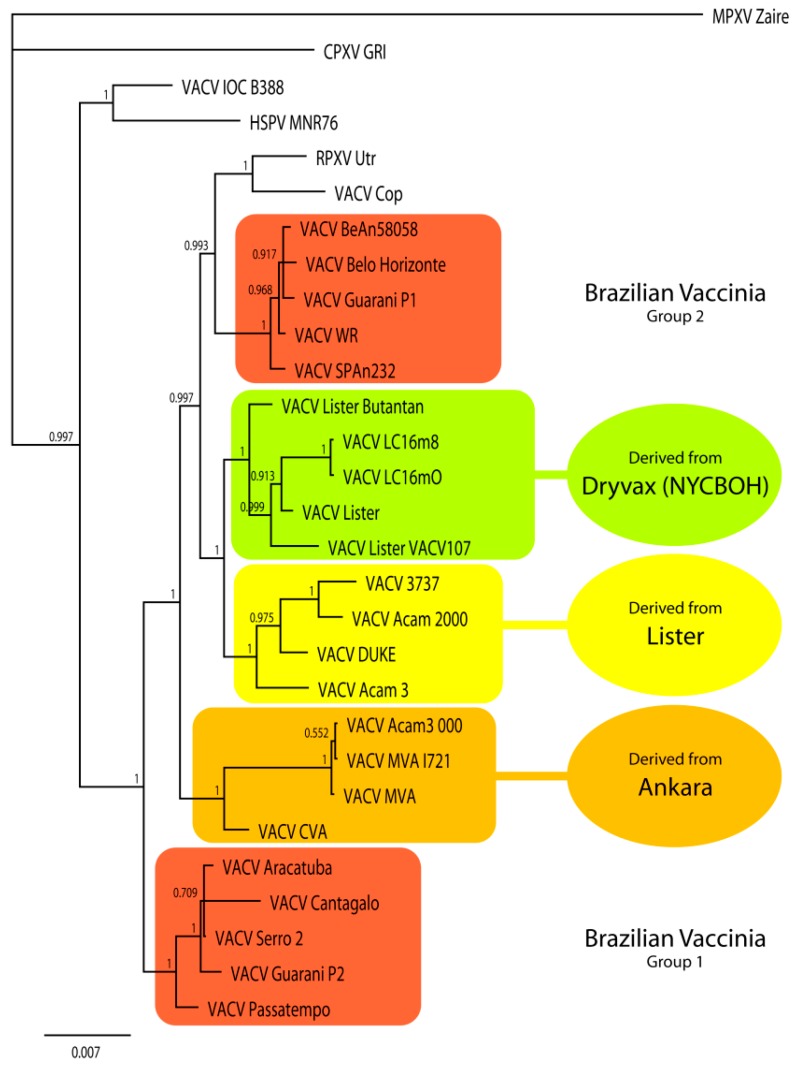
Bayesian phylogram derived from sequences of nine coding regions (*B5R*, *B8R*, *B19R*, *C6L*, *C7L*, *E3L*, *K1L*, *K2L*, and *K3L*) involved in immune modulation plus the *A56R* gene that encodes the viral hemagglutinin. Posterior probabilities label each node.

In the interest of exploring treatment options for human vaccinia virus infections, we further examined two genes that produce known targets of antiviral drug therapies for OPXV, *F13L*, and *E9L*. The F13L product is a highly conserved p37 kDa membrane protein that plays a central role in the envelopment of MV particles to produce an egress-competent form of virus particle [60,61,62]. It is the only characterized target of the compound ST-246, an orally bioavailable antiviral drug with proven efficacy against several OPXV species [61,62]. Largely conserved, only four variable amino acids were seen across VACV in the coding region of ORF *F13L* (Figure 5). S2V did not show either of the two known mutations in F13L associated with ST-246 resistance [61,62]. Another antiviral compound with potential use against OPXV infection, cidofovir, targets the DNA polymerase (E9L) of the virus and disrupts viral DNA synthesis [63]. The ORF for S2V *E9L* are also highly conserved, presenting three-amino acid differences when compared to VACV-COP and VACV-WR (data not shown). All three amino acids changes were unique to S2V among VACV and none of the mutations are known to confer resistance to cidofovir [64,65,66,67]. Therefore, S2V is likely to be susceptible to both antiviral drug treatments.

### 3.8. Genetic Diversity between the Brazilian VACV

S2V is a virus isolated during a bovine VACV outbreak. Previous data showed genetic diversity among samples of VACV isolated in Brazil; however, many isolates have not been fully sequenced [19,20]. Therefore, a limited phylogenetic analysis was performed to present the relationships between S2V and these other BVV. Phylogenetic estimation from the nine immunomodulatory genes plus the virus hemagglutinin gene resulted in a final average standard deviation of split frequencies of 0.001813. The resulting tree is depicted in Figure 5. HSPV is the first to split from the remaining group of VACV followed by VACV-IOC. Several relationships that are well understood are reflected in monophyletic groups, such as the Dryvax strains, strains derived from the Lister vaccine, and those derived from the Ankara strain. The BVV are separated into two groups. Group 1 branches from the remaining VACV first, followed by the Ankara strains. VACV-COP and RPXV-Utr are joined together and sister to BVV group 2. Sister to this grouping is a pairing of Dryvax and Lister-derived strains. Notably, VACV-WR is positioned within group 2 as seen in previous studies [20].

## 4. Discussion

This study presents biological and molecular characterizations of the first wild VACV isolated from a human in the post-smallpox era. Information on S2V provides insights on the evolutionary history, pathogenesis, host–pathogen interactions, and immunology of the BVV and other VACV. Our analysis suggests that S2V represents a distinct lineage of vaccinia virus differentiated from those derived from the Ankara, Dryvax, and Lister strains.

The in vitro and in vivo analysis showed that S2V is phenotypically different from both the prototype strain VACV-WR and the smallpox-vaccine strains used worldwide. Even though it replicates at the same rate in vitro compared to VACV-NYCBH Acambis-2000 (Figure 2B), the plaques produced in BSC40 cells are smaller than most of the VACV strains analyzed (Figure 2A). Due to its clonal derivation from VACV-NYCBH Dryvax and similar response characteristics, VACV-NYCBH Acambis-2000 was used here as a surrogate for VACV-NYCBH Dryvax itself, which is known to contain a population of variable vaccinia virus clones.

Intradermal infection in the ear pinnae of BALB/c mice showed that S2V is less virulent than Acambis-2000 and VACV-WR. Animals infected with S2V presented a tiny lesion with virtually no surrounding inflammation. Equivalent amounts of VACV Acambis-2000 or VACV-WR produced larger lesions, and WR lesions manifest with substantial surrounding induration (Figure 3). At day 5 post-infection, the peak of viral replication in this model of infection, ears of animals infected with WR had the highest viral load, followed by those from S2V and VACV Acambis-2000. The attenuated phenotype that S2V showed in mice is in contrast to the clinical findings in the human patient from whom S2V was isolated [28]. In the same way, Huemer et al. isolated a CPXV strain that proved virulent to humans and showed an attenuated phenotype in mice in a model of intranasal infection [68]. The authors of the latter work then speculated that the low virulence presented by this CPXV strain in mice would be due to the adaptation of CPXV in rodents. This hypothesis would also be applied in our case. It is worth mentioning that a Brazilian VACV isolate (*Mariana virus*) was obtained from a peridomestic rodent in an area affected by a BV outbreak and that this isolate is identical to other isolates obtained from milking cows and humans during the same outbreak, indicating that rodents might play a role as reservoirs for wild strains of VACV [12]. These apparent contradictions may eventually be understood through studies to define the “adaptation” of zoonotic VACV and CPXV to a rodent reservoir [68,69].

These findings prompted us to seek for SNPs or INDELs in the S2V genome that might provide insights as to its attenuation in mice and, at the same time, its virulence to humans [54]. In our study, although knowing that SNPs and other polymorphisms can be found amongst VACV clones from a given clinical sample, we chose to sequence only one plaque isolate to assure the best conditions for genome assembly, since the sequencing of a polyclonal population could cause ambiguity during the assembly of variable-conserved interface genomic regions. This is due to the fact that several works demonstrated that viral clones with similar plaque sizes (present in a same sample) used to have similar nucleotide content and cluster together in phylogenetic trees based on several molecular markers. Our in silico analysis showed that S2V presents a general genome organization that is similar to other VACV, but there are some differences, especially in the terminal genome regions (Supplementary Materials).

Accelerated by the possibility of deliberate VARV reintroduction and emergence of zoonotic poxviruses, recent research has centered attention on the OPXV host range, virulence, and immune protection. Applied research interest in these areas is mainly focused on the development of new diagnostic targets, safe VACV vaccines, and therapeutics. We undertook comparative analyses regarding envelope-associated, and host immune response genes of S2V to generate hypotheses to correlate the biological and clinical observations with the molecular results. Even though all of the envelope genes are present in S2V, some amino acid changes occur and might contribute to the phenotype observed. The most striking differences were found on S2V ORF *E2L*. This ORF was truncated by seven amino acids in comparison to other VACV and presented four amino acid changes, one unique to S2V, D700N (Figure 4). *E2L* is a well-conserved gene among the chordopoxviruses and it encodes a protein that is mainly found in EV particles. *E2L* null mutants are known to produce very small plaques and reduced amounts of mature virions when propagated in several cell lines [70]. The truncation and D700N mutation seen in S2V *E2L* are worthy of further analysis regarding their effects on the virus phenotype.

Recent focus on poxvirus research has inspired efforts to better understand the ability of VACV to confer immunological protection against smallpox and other zoonotic poxviruses. Previous studies have shown that some particular genes encoded by the poxvirus genome are primary targets in activating the humoral response. The *B5R* gene encodes a glycosylated enveloped virus membrane protein that is critical for the externalization of the virus and consequently cell to cell transmission and virus virulence, while also a major target for neutralizing antibodies [71]. The B5 protein is made up of a transmembrane domain and an ectodomain. The latter contains four domains with similarity to short consensus repeats (SCRs) plus a “stalk” of 51 amino acids located adjacent to the transmembrane region. Neutralizing antibodies mapped to two main regions in the protein: the first site is in the SCR1-SCR2 region and the second is in the stalk region [72]. S2V presents some amino acid changes in the stalk region (amino acids 240 and 243) that could possibly affect the neutralizing abilities of the antibodies. Recent analysis of the attenuated LC16mO strain of VACV confirmed the single base deletion in the *B5R* gene which creates a premature stop codon [73]. LC16mO strain was produced by innumerous passages of VACV-LIS strain in cell culture and has a small plaque phenotype.

OPXV encode several classes of proteins with immunomodulatory properties that have evolved to inhibit diverse processes of the host innate and adaptive immune response. The main targets are natural killer cells, cytotoxic T lymphocytes, apoptosis, complement response, chemokine production, inflammatory cytokines and the interferon system [74,75,76,77,78,79]. Like VACV-COP, the ORF *B13R* of S2V is missing approximately two thirds of the coding region seen in VACV-WR and HSPV and is likely not expressed (Table 2). This ORF codes for the serine protease inhibitor 2 (serpin 2/SPI-2), which inhibits IL-1β–converting enzyme (ICE), thereby preventing the production of mature IL-1β from pro-IL-1β. In addition, B13R is a potent inhibitor of caspase-1 and caspase-8. Therefore, the expression of B13R inhibits two important aspects of the host immune response: the processing of pro-inflammatory cytokines and apoptosis [80]. Interestingly, recombinant VACV-WR lacking the *B13R* gene caused a larger lesion than did the parental virus in the intradermal model of infection in mice. Another striking difference is present in the gene encoding the IL-1β receptor homolog of S2V (*vIL-βR*; *B16R* in VACV-COP). This protein binds to IL-1β and inhibits IL-1β-mediated fever in response to VACV infection. Smallpox vaccine strains lacking the *vIL-1βR* gene, VACV-COP for example, induced fever in murine models. In fact, VACV-COP was banned from the World Health Organization (WHO) eradication campaign because it was considered a highly virulent strain [1]. The absence of the two genes (*B13R* and *B16R*) in the S2V genome may help to explain the debilitating symptoms seen in the patient infected with this virus, which include fever, myalgia, headache, and lymphadenopathy [28]. On the other hand, in vivo over-expression of IL-1α in mice led to a faster clearance of VACV upon skin scarification infection, which may help to explain why S2V caused a lesion so small and was so rapidly cleared in our experiments of intradermal infection in BALB/c mice (Figure 3).

In spite of some variability, all poxviruses target the host immune pathway mediated by IFNs, which highlights the crucial role IFNs play in the restriction of poxviral infections. Due to this, we have looked at four genes more closely, *E3L*, *K3L*, *B8R*, and *B19R*, which are related to the IFN response interfering with the IFN pathway or encoding IFN viroceptors [81]. All genes are present in the S2V genome and their structure appear to be intact and similar to that of VACV-COP and WR (Figure 4 and Table 2) (data not shown). This finding could explain the marked immunomodulation seen in PBMCs isolated from the patient infected with S2V, which presented a down modulation of the activation marker cluster of differentiation (CD) 25 and lower IFN-γ production after stimulation with inactivated VACV antigens [28].

To reconstruct the evolutionary history of S2V, genes related to immunomodulation, besides the canonical phylogenetic target, *A56R* gene, were aligned and concatenated to perform a phylogenetic analysis. This analysis corroborates several known relationships between VACV strains and suggests an early divergence of the BVV group 1, including S2V. As described previously, the BVV strains grouped into two well-defined clades which do not appear as each other’s closest relatives [19,20]. A recent study, using a genome-wide approach, grouped S2V a Brazilian VACV lineage with Cantagalo virus (CTGV) and the vaccine strain IOC [82]. However, the lack of additional genome sequences of Brazilian feral samples hampers a conclusive statement about their origins. In our analyses, S2V grouped with other Brazilian VACV lineages, as previously reported [9,10,11,12,20,83], while the VACV-IOC strain was grouped in a distinct branch with HSPV. Our data supports the circulation of two distinct Brazilian VACV lineages, possibly with distinct evolutionary history.

The low virulence phenotype of S2V in mice (Figure 3) is in agreement with phenotypes reported for other BVV strains of the same group [22]. BVV group 2 is a cluster of more virulent isolates, including the mouse neurovirulent laboratory strain VACV-WR. This topology could be a reflection of parallel evolution of the virulence genes used to construct the tree. Additional genome sequencing of BVV strains will be critical to understanding the origins of VACV circulating in Brazil.

## Figures and Tables

**Table 1:** Genomes and sequences used in this study.

Virus Species	Strain (Abbreviation)	GenBank Accession Number(s)
Cowpox virus	GRI-90 (CPXV-GRI)	X94355
Monkeypox virus	Zaire-96-I-16 (MPXV Zaire96)	AF380138
Vaccinia virus	3737 (VACV-3737)	DQ377945
	Acambis-2000 (VACV Acam2000)	AY313847
	Acambis-3000 (VACV-Acam3000)	AY603355
	Acambis clone 3 (VACV-Acam3)	AY313848
	Araçatuba	EF051269, EF051277, EF051285, EF175987, EF175965, DQ194389, AY523994, EF175973, DQ194382
	BeAn-58058	EF051270, EF051278, EF051286, EF175990, EF175968, DQ194388, DQ206442, EF175976, AF261890
	Belo Horizonte	EF051276, EF051284, EF051292, EF175993, EF175971, DQ194390, DQ206435, EF175979, DQ194383
	Cantagalo ^#^	KT013210
	Chorioallantois Vaccinia Ankara (VACV-CVA)	AM501482
	Copenhagen (VACV-COP)	M35027
	Duke (VACV-DUKE)	DQ439815
	Guarani P1	EF051271, EF051279, EF051287, EF175991, EF175969, DQ194385, DQ206436, EF175977, DQ194380
	Guarani P2	EF051272, EF051280, EF051288, EF175988, EF175966, DQ194386, DQ206437, EF175974, DQ194381
	Horsepox virus (HSPV-MNR76)	DQ792504
	IOC Brazil ^#^ (VACV-IOC-B388)	KT184691
	Lister 107 France	DQ121394
	Lister Butantã * Brazil	EF175981, EF175982, EF175983, EF175994, EF175972, EF175984, EF175985, EF175980
	Lister Japan (VACV-Lister)	AY678276
	Lister LC16m0 (VACV-LC16m0)	AY678277
	Lister LC16m8 (VACV-LC16m8)	AY678276
	Western Reserve (VACV-WR)	AY243312
	Modified Vaccinia Ankara (VACV-MVA)	U94848
	Modified Vaccinia Ankara 1721 (VACV-MVA-1721)	DQ983236
	Passatempo	EF051274, EF051282, EF051290, EF175989, EF175967, DQ530240, DQ070848, EF175975, DQ530239
	Rabbitpox virus Utrecht (RPXV-Utr)	AY484669
	Serro2 (S2V)	KF179385
	SpAn232	EF051283, EF051291, EF175992, EF175970, DQ194387, DQ222922, EF175978, DQ194384, EF051275

* VACV Lister Butantã does not have a *B19R* gene; ^#^ Only eight (*B19R*, *B8R*, *K1L*, *K2L*, *C7L*, *E3L*, *A56R*, and *K3L*) of the nine genes analyzed (*A56R*, *B8R*, *B19R*, *C6L*, *C7L*, *E3L*, *K1L*, *K2L*, and *K3L*) are available in GenBank for Cantagalo Virus (CTGV). The *C6L* gene of VACV-IOC is annotated as two open reading frames (ORFs) (VACV_IOC_B388_035 and 036); both were used in the alignment.

**Table 2:** VACV immunomodulatory genes investigated in this study.

Gene	Viral Product	Characteristic/Function	Coding Region in S2V
*A46R*	Blocks TLR-mediated signaling	Antagonizes TLR signaling. Inhibits NF-κB activation. Blocks IFN response.	Present
*A52R*	Blocks TLR-mediated signaling	Antagonizes TLR signaling. Blocks NF-κB activation by multiple TLRs and associates with IRAK2 and TRAF6. Blocks IFN response.	Present
*A53R*	Binds to TNF-α	CrmC. Viroceptor. Secreted TNF inhibitor. TNF receptor homolog.	Present
*B19R*	IFNα/β receptor homolog	Viroceptor. Mimics IFNα/β receptor. Binds and inhibits the activity of type I IFN. B18R in VACV-WR.	Present
*B8R*	IFNγ receptor	Viroceptor. Mimics IFNγ receptor. Binds and inhibits the activity of type II IFN.	Present
*B13R*	Serpin-1, -2, -3 gene family (SPI-2/CrmA)	Inhibits IL-1 converting enzyme (caspase).	Present; truncated as in VACV-COP
*B16R*	IL-1β receptor homolog	Viroceptor. Blocks febrile response in a poxvirus infection. B15R inVACV-WR.	Early stop codon
*C3L*	Complement control protein	Virokine. Inhibits the classical and alternative complement activation pathways.	Present
*C7L*	Antiapoptotic protein	Apoptosis inhibitor; host range virulence factor.	Present
*C12L*	IL-18 binding protein	Virokine. Natural antagonist of IL-18. Inhibits IL-18 induced IFN-γ production.	Present
*C11R*	Vaccinia growth factor	Virokine. Stimulates cell growth. Virulence factor.	Present
*E3L*	dsRNA binding protein	IFN inhibitor. Antiapoptotic protein. Sequesters dsRNA and prevents activation of PKR and OAS.	Present
*K3L*	eIF-2α mimic	Antiapoptotic protein. Mimics eIF-2α and prevents activation of PKR.	Present
*F1L*	Mitochondrial-localized protein	Virokine. Protects cells from apoptotic death and inhibits cytochrome c release. Antiapoptotic protein.	Present
*K1L*	Host range protein	Virokine. Blocks signaling pathway for NF-κB activation. Inhibits proinflammatory genes expression.	Present
*M2L*	NF-κB inhibitor	Antiapoptotic factor.	Present
*N1L*	Antiapoptotic protein	Virokine. Blocks signaling pathway for NF-κB activation by TNF.	Present

Gene nomenclature is based on VACV-COP. This table was constructed based on information provided by recent literature. eIF-2α: eukaryotic initiation factor-2α; dsRNA: double-stranded RNA; IFN: interferon; IL: interleukin; IRAK2: interleukin-1 receptor-associated kinase-like 2; NF-κB: nuclear factor κ-light-chain-enhancer of activated B cells; OAS: 2′-5′ oligoadenylate synthetase; PKR: protein kinase R; serpin/SPI: serine protease inhibitor; TLR: Toll-like receptor; TNF: tumor necrosis factor; TRAF6: TNF receptor-associated factor 6.

## Supplementary Materials

The following are available online at www.mdpi.com/1999-4915/8/12/328/s1, Figure S1: Vaccinia virus (VACV) strain Serro 2 virus (S2V) open reading frame (ORF) map; Table S1: VACV immunomodulatory genes investigated in this study; Table S2: Genomes and sequences used in this study.
