# Supplementary material for: Serro 2 Virus Highlights the Fundamental Genomic and Biological Features of a Natural Vaccinia Virus Infecting Humans
Source: Viruses. 2016 Dec 10;8(12):328. doi: 10.3390/v8120328 (PMC5192389; doi:10.3390/v8120328)
Supplement: Supplementary file 1 [file viruses-08-00328-s001.docx]

Supplementary Materials: Serro 2 Virus Highlights the Fundamental Genomic and Biological Features of a Natural Vaccinia Virus Infecting Humans

Giliane de Souza Trindade, Ginny Emerson, Scott Sammons, Michael Frace, Dhwani Govil, Bruno Eduardo Fernandes Mota, Jônatas Santos Abrahão, Felipe Lopes de Assis,
Melissa Olsen-Rasmussen, Cynthia S. Goldsmith, Yu Li, Darin Carroll, Flavio Guimarães da Fonseca, Erna Kroon and Inger Damon


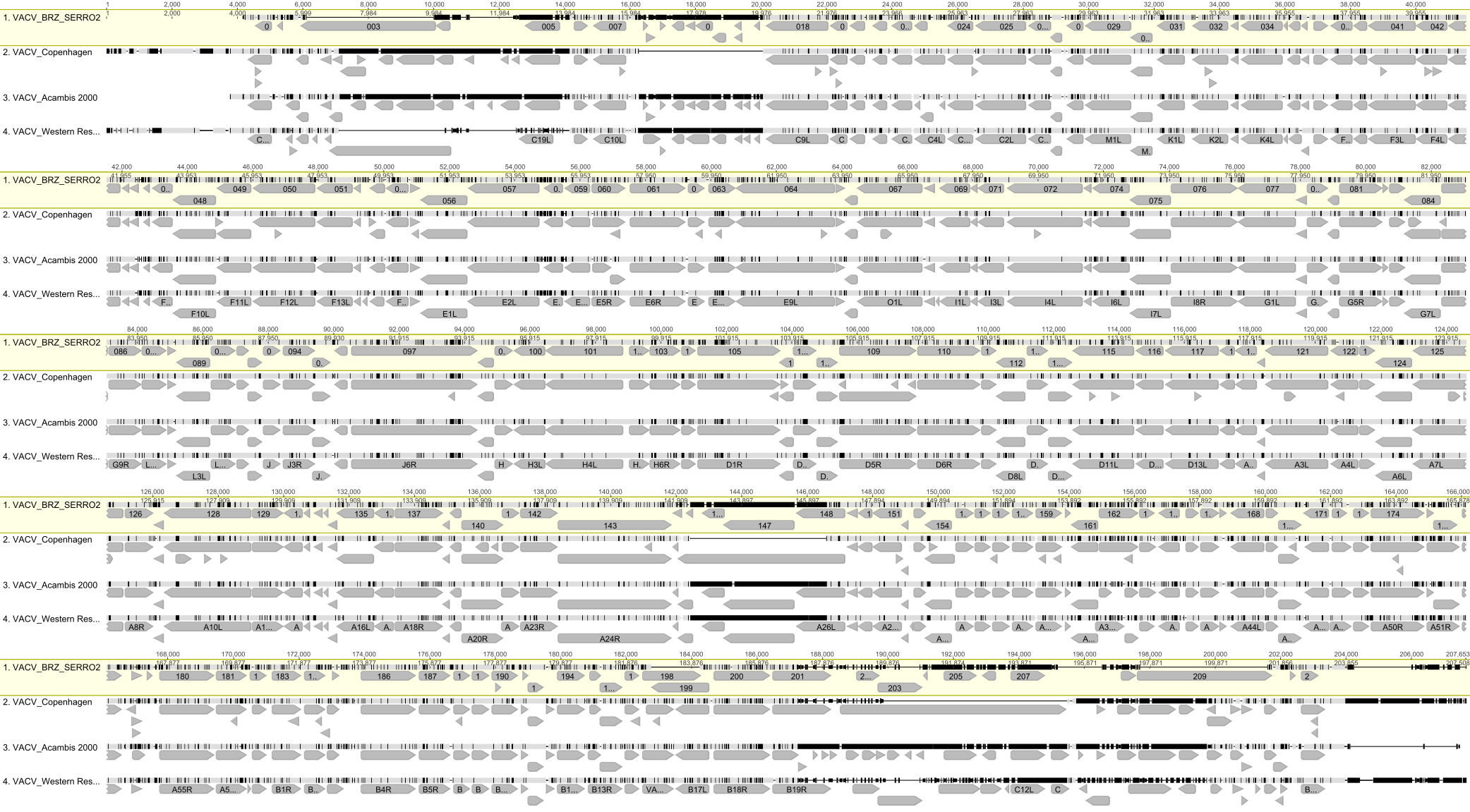


**Figure S1.** Vaccinia virus (VACV) strain Serro 2 virus (S2V) open reading frame (ORF) map. This map shows a comparison between the whole genome of S2V,
VACV- Copenhagen (COP) (reference strain), and VACV-Acambis-2000. The S2V genome is about 185 kbp in length and has a G/C content of 33%.

**Table S1.** VACV immunomodulatory genes investigated in this study.

| **Gene** | **Viral Product** | **Characteristic/Function** | **Coding Region in S2V** |
| --- | --- | --- | --- |
| *A39R* | Gene fragment, semaphorin-like | Host defense modulator. Proinflammatory protein in VACV-mouse skin lesion model. | Present |
| *A40R* | C-type lectin-like type-II membrane protein | Host defense modulator. Deletion attenuates intradermal lesion in VACV-mouse model | Present |
| *A41L* | Secreted glycoprotein | Host defense modulator. Deletion causes more severe lesion and enhanced viral clearance in VACV-mouse skin model. | Present |
| *A44L* | Hydroxysteroid dehydrogenase | Host defense modulator. Deletion attenuates intradermal lesion in VACV-mouse model | Present |
| *A46L* | Block TLR-mediated signaling | Antagonizes TLR signaling. Inhibits NF-κB activation. Blocks IFN response. | Present |
| *A52R* | Block TLR-mediated signaling | Antagonizes TLR signaling. Blocks NF-κB activation by multiple TLRs and associates with IRAK2 and TRAF6. Blocks IFN response. | Present |
| *A53R* | Binds to TNF-α | CrmC. Viroreceptor. Secreted TNF inhibitor.TNF receptor homolog. | Present |
| *B19R* | IFN-α/β receptor homolog | Viroreceptor. Mimics IFN-α/β receptor. Binds and inhibits the activity of type I IFN. B18R in  VACV-WR. | Present |
| *B8R* | IFN-γ receptor | Viroreceptor. Mimics IFN-γ receptor. Binds and inhibits the activity of type II IFN. | Present |
| *B13R* | Serpin-1, -2, -3 gene family  (SPI-2/CrmA) | Inhibits IL-1 converting enzyme (caspase) | Present; truncated-like VACV-COP |
| *B16R* | IL-1β receptor homolog | Viroreceptor. Blocks febrile response in a poxvirus infection. B15R inVACV-WR. | Early stop codon |
| *B29R* | ITR chemokine-binding protein | Host defense modulator. | Early stop codon |
| *C3L* | Complement control protein | Virokine. Inhibits the classical and alternative complement activation pathways. | Present |
| *C7L* | Antiapoptotic protein | Apoptosis inhibitor; host range virulence factor. | Present |
| *C12L* | IL-18 binding protein | Virokine. Natural antagonist of IL-18. Inhibits IL-18 induced IFN-γ production. | Present |
| *C11R* | Vaccinia growth factor | Viral cytokine. Stimulates cell growth.  Virulence factor. | Present |
| *C23L* | ITR chemokine-binding protein | Host defense modulator. | Early stop codon |
| *E3L* | dsRNA binding protein | IFN inhibitor. Antiapoptotic protein. Sequesters dsRNA and prevents activation of PKR and OAS. | Present |
| *K3L* | eIF-2α mimic | Antiapoptotic protein. Mimics eIF-2α and prevents activation of PKR. | Present |
| *F1L* | Mitochondrial- localized protein | Virokine. Protect cells from apoptotic death and inhibits cytochrome c release. Antiapoptotic protein. | Present |
| *K1L* | Host range protein | Virokine. Blocks signaling pathway for NF-κB activation. Inhibits proinflammatory  gene expression. | Present |
| *M2L* | NF-κB inhibitor | Anti-apoptotic factor. | Present |
| *N1L* | Antiapoptotic protein | Virokine. Blocks signaling pathway for NF-κB activation by TNF. | Present |

Genes nomenclature based on VACV-COP strain. This table was constructed based on information provided by some recent literature.

**Table S2.** Genomes and sequences used in this study.

| **Virus Species** | **Strain (Abbreviation)** | **GenBank Accession Number(s)** |
| --- | --- | --- |
| Cowpox virus | Austria 1999  (CPXV-AUS 1999 867) | HQ407377 |
|  | GRI-90 (CPXV-GRI) | X94355 |
| Monkeypox virus | Zaire-96-I-16 (MPXV Zaire96) | AF380138 |
| Vaccinia virus | 3737 (VACV 3737) | DQ377945 |
|  | Acambis-2000  (VACV-Acam2000) | AY313847 |
|  | Acambis-3000  (VACV-Acam3000) | AY603355 |
|  | Acambis clone 3  (VACV-Acam3) | AY313848 |
|  | Araçatuba | EF051269, EF051277, EF051285, EF175987,EF175965,  DQ194389, AY523994, EF175973, DQ194382 |
|  | BeAn 58058 | EF051270, EF051278, EF051286, EF175990, EF175968,  DQ194388,DQ206442, EF175976, AF261890 |
|  | Belo Horizonte | EF051276, EF051284, EF051292, EF175993, EF175971,  DQ194390, DQ206435, EF175979, DQ194383 |
|  | Cantagalo | AY500815, EF488961, EF488959, AY771338, AF229247, AY772447, EU003185, EU528619 |
|  | Chorioallantois Vaccinia Ankara (VACV-CVA) | AM501482 |
|  | Copenhagen (VACV-COP) | M35027 |
|  | Duke (VACV-DUKE) | DQ439815 |
|  | Guarani P1 | EF051271, EF051279, EF051287, EF175991, EF175969,  DQ194385, DQ206436, EF175977, DQ194380 |
|  | Guarani P2 | EF051272, EF051280, EF051288, EF175988, EF175966,  DQ194386, DQ206437, EF175974, DQ194381 |
|  | Horsepox virus (HSPV MNR76) | DQ792504 |
|  | IOC Brazil (VACV-IOC) | AF229248, EU528618, EU003186, AY500816, EF488966, EF488964, AY772448, DQ070236 |
|  | Lister 107 France | DQ121394 |
|  | Lister Butantã Brazil | EF175981, EF175982, EF175983, EF175994, EF175972,  EF175984, EF175985, EF175980 |
|  | Lister Japan (VACV Lister) | AY678276 |
|  | Lister LC16m0 (VACV LC16m0) | AY678277 |
|  | Lister LC16m8 (VACV LC16m8) | AY678276 |
|  | Western Reserve (VACV-WR) | AY243312 |
|  | Modified Vaccinia Ankara (VACV-MVA) | U94848 |
|  | Modified Vaccinia Ankara 1721 (VACV MVA 1721) | DQ983236 |
|  | Passatempo | EF051274, EF051282, EF051290, EF175989, EF175967, DQ530240, DQ070848, EF175975, DQ530239 |
|  | Rabbitpox virus Utrecht  (RPXV-Utr) | AY484669 |
|  | Serro2 (SV2) | M35027 |
|  | SpAn232 | EF051283, EF051291, EF175992, EF175970, DQ194387, DQ222922, EF175978, DQ194384, EF051275 |
